# Electrical Neuroimaging of Music Processing in Pianists With and Without True Absolute Pitch

**DOI:** 10.3389/fnins.2019.00142

**Published:** 2019-03-13

**Authors:** Sélim Yahia Coll, Noémi Vuichoud, Didier Grandjean, Clara Eline James

**Affiliations:** ^1^Neuroscience of Emotion and Affective Dynamics Laboratory Faculty of Psychology and Educational Sciences and Swiss Centre for Affective Sciences, University of Geneva, Geneva, Switzerland; ^2^School of Health Sciences Geneva HES-SO University of Applied Sciences and Arts Western Switzerland, Geneva, Switzerland; ^3^Geneva Neuroscience Center University of Geneva, Geneva, Switzerland

**Keywords:** complex music, electrical source imaging, ERP microstate analysis, trained pianists, true absolute pitch

## Abstract

True absolute pitch (AP), labeling of pitches with semitone precision without a reference, is classically studied using isolated tones. However, AP is acquired and has its function within complex dynamic musical contexts. Here we examined event-related brain responses and underlying cerebral sources to endings of short expressive string quartets, investigating a homogeneous population of young highly trained pianists with half of them possessing true-AP. The pieces ended regularly or contained harmonic transgressions at closure that participants appraised. Given the millisecond precision of ERP analyses, this experimental plan allowed examining whether AP alters music processing at an early perceptual, or later cognitive level, or both, and which cerebral sources underlie differences with non-AP musicians. We also investigated the impact of AP on general auditory cognition. Remarkably, harmonic transgression sensitivity did not differ between AP and non-AP participants, and differences for auditory cognition were only marginal. The key finding of this study is the involvement of a microstate peaking around 60 ms after musical closure, characterizing AP participants. Concurring sources were estimated in secondary auditory areas, comprising the planum temporale, all transgression conditions collapsed. These results suggest that AP is not a panacea to become a proficient musician, but a rare perceptual feature.

## Introduction

The present study applied electrical neuroimaging to investigate the influence of true absolute pitch on processing of complex classical music in pianists trained in the western classical repertoire. The research is a follow-up of 3 previous studies that showed progressive processing changes with musical training intensity and proficiency using the same musical stimuli (Oechslin et al., 2013; James et al., 2017; Jenni et al., 2017) and allows to answer the question whether true-absolute pitch offers additional advantages for music processing.

Absolute pitch (AP) perception is the rare faculty to identify or produce instantaneously any musical tone or other sound according to the 12-tone equal temperament, in the strictest sense with semitone precision, without external reference (Miyazaki, 1988; Takeuchi and Hulse, 1993; Levitin and Rogers, 2005; Elmer et al., 2015). In AP possessors, this tone identification is performed automatically and cannot be suppressed (Akiva-Kabiri and Henik, 2012).

Levitin and Rogers (2005) propose an alternative more clement model of “latent” or quasi-absolute pitch (QAP). These authors could show that almost half of the non-musician Western population is able to sing pitches of familiar pop songs from memory with at least whole tone precision (Levitin, 1994). Although this finding is highly interesting in the context of musicality as a human universal, the current research focused exclusively on semitone precision AP with very high accuracy. This extreme or “true” form of AP also called AP-1 (Baharloo et al., 1998, 2000) or “genuine” AP (Bachem, 1937, 1955) constitutes a distinct and truly exceptional capacity, with an “all-or-nothing” quality (Profita and Bidder, 1988) that should be distinguished from QAP. Moreover, true-AP could be anatomically dissociated from both Relative Pitch (RP) and QAP, with larger left-right planum temporale asymmetry in true-AP possessors (Wilson et al., 2009).

Except for tone-deaf amusics (Ayotte et al., 2002; Tillmann et al., 2015), the rest of the population rather relies on RP and processes musical idiom contextually, comparing tones with surrounding ones. However, according to recent observations (Wengenroth et al., 2014; Ziv and Radin, 2014), both AP and RP capacities can be considered as continuous distributions, with a distinctive independent position in both distributions for each individual. So, in the end, any normal individual possesses both AP and RP to some extent (Zatorre, 2003; Wengenroth et al., 2014; Ziv and Radin, 2014).

Whether possessing AP is an advantage or even the panacea to become an exceptional musician is a matter of debate. AP can represent an advantage for developing musical abilities by strengthening internal representations, facilitating musical memory and for score reading, musical dictation or composing (Dooley and Deutsch, 2010). However, the faculty can also hamper proper functioning, as the automatic AP response may cause interference in the context of special tunings, ensemble playing or transpositions (Miyazaki and Rakowski, 2002; Zatorre, 2003; Wilson et al., 2009). According to Ziv and Radin (2014), AP may increase reaction times in the context of global processing of music as opposed to local processing.

AP only develops in those who receive musical tonal training from an early and before a critical age (Gregersen et al., 2001; Vitouch, 2003). As a consequence, classically reported percentages of AP in the general population (0.01%; Bachem, 1955; Ward, 1999) are probably underestimated, as no consensus exists on how to appraise latent AP in people lacking musical education. Finally, training is not sufficient to develop AP, which is also a function of genetic predisposition, often occurring in families (Baharloo et al., 2000; Zatorre, 2003). The estimated proportion is larger in highly trained Western musicians (Baharloo et al., 1998; Gregersen et al., 1999; up-to 15% and over), and much larger in Chinese musicians speaking Mandarin, a tone language (up-to 60% with semitone precision; Deutsch et al., 2006). These observations probably reflect the influence of intensive tonal experience from birth on AP acquisition. However, some influence of genetic factors cannot be excluded in Asian populations (Gregersen et al., 1999).

Some authors propose a 2-level model of AP functioning (Levitin, 1994; Zatorre, 2003; Levitin and Rogers, 2005). The first level involves the representation of pitches in long-term memory and is essentially perceptual in nature. The second level, principally cognitive, involves the categorization or labeling of pitch according to the 12-tone temperament. These 2 levels may rely on distinct cerebral networks (Elmer et al., 2015). The left planum temporale might foster long-term pitch representation, allowing matching incoming spectro-temporal patterns with a template (Ohnishi et al., 2001; Griffiths and Warren, 2002). As a matter of fact, degree of left planum temporale activity correlated significantly with absolute pitch ability (Ohnishi et al., 2001). The left posterior dorsolateral prefrontal cortex would then perform the pitch-verbal associations (Zatorre et al., 1998). The bridge between these networks may be assured by functional connectivity in the left hemisphere that could be shown by theta band synchronization, likely via the arcuate fasciculus (Elmer et al., 2015). Wengenroth et al. (2014), using a unique AP test excluding the intervention of RP during the AP task, rather found an association between AP and the volume of the right Heschl's gyrus and proposed that a right-hemispheric network mediates AP perception. However, this study also accepted whole tone precision -albeit with weighted scoring-, and did thus not focus exclusively on true-AP. A recent publication explained observed differences concerning AP by the diversity of its definitions, and the lack of consensus concerning the tasks used to measure this faculty (Hou et al., 2016). In the current experiment we focused exclusively on true-AP possessors as compared to all other forms of pitch processing.

Most research on auditory processing in AP and non-AP (NAP) populations focused on the processing of isolated tones, chords or on oddball paradigms (McLachlan et al., 2013; Wengenroth et al., 2014; Ziv and Radin, 2014; Rogenmoser et al., 2015; Greber et al., 2018). However, it is in the context of complex dynamic musical contexts that AP is acquired and has its function. In the current study, we examined event-related brain responses to expressive polyphone musical stimuli, investigating a homogeneous population of young highly trained pianists with half of them possessing true-AP.

The musical stimuli were short expressive pieces for string quartet with 3 levels of harmonic transgression at closure that participants appraised, presented while high density EEG was recorded. We previously used these stimuli successfully to show the impact of different levels of musical training intensity on brain and behavioral responses using fMRI (Oechslin et al., 2013) and EEG (James et al., 2017), also exclusively in pianists. Both the fMRI and the EEG study results disclosed progressive changes in music processing as a function of musical training intensity and proficiency. The present study used EEG neuro-imaging to investigate whether differences in processing of these pieces would also occur as a function of absolute pitch possession within a homogeneous population of highly trained pianists. EEG neuro-imaging allowed to compare AP and NAP for Event Related Potentials (ERPs), functional ERP microstates and underlying brain sources (Michel and Murray, 2012), providing millisecond precision information on the time course of brain processing.

Microstates are stable voltage topographies over time, lasting tens to hundreds of milliseconds, reflecting time windows of coherent synchronized activation of large-scale neuronal networks that represent basic physiological units of cognition (Lehmann et al., 1987; Murray et al., 2008; Brunet et al., 2011; James et al., 2017). Microstate analysis or spatio-temporal ERP analysis, compared to the isolated amplitude analysis of an array of single electrode sites, has the advantage of conserving the major part of the variance in the data by extracting the microstates in a multivariate way including all electrodes, groups and conditions in one unified analysis (Brunet et al., 2011; Michel and Murray, 2012; James et al., 2017). Classical ERP analysis, studying an array of isolated ERPs, although most effective in differentiating experimental conditions and groups at specific electrode sites (Kutas and Federmeier, 2011; Koelsch et al., 2013), only explores a small part of the variance in the data and does not explore the spatial dimensions of high-density EEG (Michel and Murray, 2012). Especially in the context of intrinsically different populations like in the current study, studying all electrodes and their underlying sources provides a more comprehensive view on the functioning of the brain. Moreover, changes in the spatial configuration of the microstates, indicate a change in the underlying cerebral sources (Vaughan, 1982; Michel and Murray, 2012), making them reliable precursors for EEG source imaging, thus allowing comparisons with other neuroimaging results. Although source localizations via EEG only allow centimeter precision, the combined temporal and spatial analyzes may shed new light on the time course of cerebral processing. Rigorous statistical thresholds may ensure reliability of these computations.

Some indications exist for possible cognitive advantages of AP outside the music domain, particularly for auditory span or short-term memory (STM; Deutsch and Dooley, 2013). In order to verify the possible influence of true-AP on general cognition, we also compared measures of auditory STM and working memory (WM) as well as the results of an auditory figure-ground perception (“hearing in noise”) test between the groups.

This experimental plan allowed us to examine the following hypothesis.

We anticipated that AP musicians would outperform NAP musicians for harmonic transgression sensitivity, following their automatic and infallible pitch analysis (Hypothesis 1), and that specific ERP patterns and microstates would occur in response to the harmonic transgressions. As this has never been investigated before in this population in a dynamic ongoing musical context, we cannot foresee the type of ERPs that may occur.

We expected both early perceptual differences in the ERPs and later more cognitive differences (Hypothesis 2), reflecting the 2-level model of AP functioning (Elmer et al., 2015).

We predicted influence of AP on general auditory cognition, with advantages for short term and working memory confirming the results of Deutsch and Dooley (2013) and possibly disadvantages for hearing in noise, as AP musicians automatically process all pitches (Hypothesis 3).

Finally, we hypothesized to find distinct brain sources, possibly early in time comprising the left planum temporale and later on in the prefrontal cortex in AP musicians (Hypothesis 4; Ohnishi et al., 2001).

## Materials and Methods

### Participants

Twenty-four right-handed pianists accepted to participate in this study on a voluntary basis. They granted written informed consent, and received financial compensation after participation. The pianists were recruited from the Conservatoires of Geneva, Lausanne, and Neuchâtel. Among them 12 possessed true-AP (3 men; *M* = 24.18 years, *SD* = 4.91) and 12 did not (6 men; *M* = 24 years, *SD* = 4.81; see the Results section “Absolute Pitch Test” below for mean scores and confidence intervals of both groups). One AP participant was eliminated due to EEG artifacts. Final analyses were made on 11 true-AP and 12 NAP participants. Most of them also practiced other instruments or sang, 9 in the AP group and 7 in the NAP group. Secondary practices concerned mainly wind instruments and singing, but not string instruments. As tested by Student independent sample *t*-tests and chi-squared tests for independence (Table 1), true-AP and NAP participants did not statistically differ for age, gender, socio-economic status, age onset of musical practice, total number of years of piano training, instrumental level on the piano, practice of secondary instruments and years of practice on the secondary instruments.

**Table 1:** Biographical data.

	**AP**	**NAP**	***t***/χ^2^**(df)**	***P***
Age	24.18 (5.15)	24 (5.03)	*t*_(21)_ = −0.09	0.93
Age onset of piano practice	6.86 (3.67)	7.29 (3.32)	*t*_(21)_ = 0.29	0.77
Total number of years of piano training	17.14 (7.87)	15.88 (3.86)	*t*_(21)_ = −0.49	0.63
Gender	Men: 3 Women: 8	Men: 6 Women: 6	*χ^2^*(1) = 1.24	0.26
Instrumental level	Certificated amateur: 5 Preprofessional: 4 Professional: 2	Certificated amateur: 8 Preprofessional: 4 Professional: 0	*χ^2^*(2) = 4.23	0.16
Socio-economic status	Compulsory school: 0 Matura/Baccalaureate: 2 University/Higher education: 9	Compulsory school: 1 Matura/Baccalaureate: 2 University/Higher education: 9	*χ^2^*(2) = 2.65	0.27
Practice of secondary instruments	Yes: 9 No: 2	Yes: 7 No: 5	*χ^2^*(1) = 1.50	0.22
Total number of years of secondary instruments' practice	7 (5.74)	11.64 (7.70)	*t*_(14)_ = 1.39	0.19

*Listed are the means of the variables of both groups and the p-values (t-tests for the quantitative variables and chi-square test for independence for the qualitative variables)*.

*SDs are reported in brackets*.

Concerning the instrumental level (see Table 1), 13 pianists (5 AP, 8 NAP) obtained a certificate from the amateur sections of the above-mentioned Conservatoires, thus reaching the highest amateur level. The certificate implies up to 14 years of piano instruction, public final exams, 9 years of music theory closed by examinations, and 3 years of supplementary music instruction like composition or chamber music. The 8 pre-professionals (4 AP and 4 NAP) also obtained the certificate and were admitted in the preparatory classes of the Conservatoires and expected to enter the professional section in maximum 3 years. Finally, 2 AP participants already entered the professional section of one of the Conservatoires. No significant difference existed between the instrumental levels of AP and NAP participants [see Table 1; χ^2^(2) = 4.23, *P* = 0.16].

In the current experiment, the musical proficiency of the participants was confirmed by their elevated d-prime scores for detecting both apparent and subtle transgressions in the musical test (see section “Musical Test”).

All participants reported normal hearing and presented no history of neurological illnesses. The experiment was conducted at the Brain and Behavior Laboratory in the “Centre Médical Universitaire” in Geneva and approved by the local ethics committee.

### Materials and Trial Sequence

Participants realized 6 tasks described in order of execution below.

#### Edinburgh Handedness Inventory

Developed by Oldfield (1971) this questionnaire precisely assessed the laterality of each participant, providing a score between −100 (100% left-handed) and 100 (100% right-handed). This inventory comprises questions concerning handedness preference for 10 tasks, like writing or drawing.

#### Absolute Pitch Test

Created by Oechslin et al. (2010), this test is composed of 36 piano tones (*A* = 440 Hz), all repeated 3 times. Sinus tones are reported to be harder to detect than piano tones (Baharloo et al., 1998). However, Baharloo et al. found very similar scores in true-AP people for pure tones vs. piano tones. This can be explained by the “all-or-nothing” quality of pitch labeling of true-AP possessors (Profita and Bidder, 1988), for whom the timbre of the stimuli will not impact their pitch perception. Moreover, we consider piano tones to possess higher ecological validity (Elmer et al., 2013) and therefore more appropriate in this study on music processing. The resulting 108 tones were presented in pseudo-random order, with 18 Ω Klipsch Image S4 earphones. The tones were situated in the third, fourth and fifth piano octave reaching from C3 to H5. All 12 semitones of each octave were presented. Tones lasted 1 s and were separated by a 4 s inter-stimulus interval of brown noise. Tones and brown noise were generated using the “Cool Edit Pro” software (http://cool-edit-pro.soft32.com/). After each tone, participants immediately wrote down its exact label (with semitone precision), during the following 4 s window of brown noise. Each correct answer counted for 1 point, allowing a maximum score of 108 points.

At first, we recruited participants based on auto-reporting of semitone precision AP or the absence of it. For the final composition of the groups, after testing for AP, we retained the NAP group participants only if their score respected an upper limit of maximum 65% of correct answers for the 95% CI (Confidence Interval). This threshold was chosen as this percentage corresponds to the level at which adult individuals without AP may temporarily bring their semitone precision detection of tones or learn to identify one single tone following intensive training (Brady, 1970; Ward, 1999; Van Hedger et al., 2015). For the true-AP group participants, the lower limit of the 95% CI was set to 90% (Baharloo et al., 1998). Based on previous studies, AP participants who scored more than 90% with semitone precision on an AP test appeared to be a special group (Miyazaki, 1988; Baharloo et al., 1998; Itoh et al., 2005; Schulze et al., 2009). The other AP possessors that were called “mid AP” (Itoh et al., 2005) had scores around 60% on an AP test but they never attained more than 90% of corrects answers. Therefore, with a lower limit of the 95% CI fixed at 90% of correct answers for our AP group we can be confident that it represents true-AP possessors (for the sake of simplicity, from now on AP in the text).

#### Musical Test

**Figure 1:**
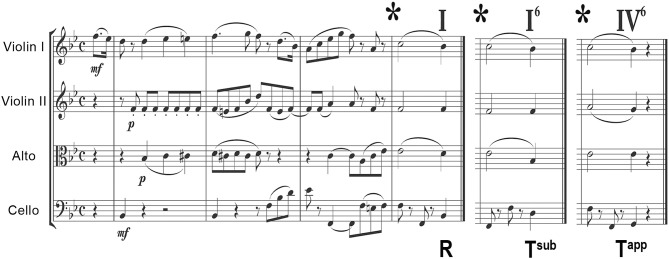
Example of a stimulus used in the musical test, a string quartet in B-flat major. The 3 different closures are shown: R corresponds to a regular ending (degree I), T^sub^ to a subtle transgression (degree I^6^) and T^app^ to an apparent transgression (degree IV^6^). Matching sound-files are provided in the Supplementary Information. For more examples, we refer to Oechslin et al. (2013), James et al. (2017), and Jenni et al. (2017).

While recording high density EEG (electroencephalogram), 90 different pieces for string quartet (2 violins, 1 alto and 1 cello), with a 10 s mean duration, were presented via EEG compatible earphones (Etymotic Research, Elk Grove Village, IL, USA) to all participants. We used string quartets in order to cancel out the “own instrument effect”, consisting in enhanced cortical responses to the timbre of a well-mastered instrument (Pantev et al., 2001; Margulis et al., 2009). A professional composer (see acknowledgments) developed the musical pieces specifically for our experiments, equally distributed over all 24 minor and major tonalities. The “Sibelius” (http://www.sibelius.com/) and “Logic Pro” (http://www.apple.com/logic-pro/) software served to create the compositions; natural instruments' timbres were implemented from the “Garritan Personal Orchestra” plug-in (http://www.garritan.com/). Musical styles of the pieces ranged from the Baroque to the Romantic period. The 90 original pieces were presented each with 3 distinct versions of the ending of the piece (see Figure 1), resulting in 270 stimuli. These 3 versions represent the 3 levels of our experimental condition “Transgression”: (1) tonic chord (I; regular ending, no transgression = R), (2) first inversion of the tonic chord (I^6^; subtle transgression = T^sub^) and (3) first inversion of the sub-dominant chord (IV^6^; apparent transgression = T^app^). Participants appraised whether closure of the pieces was appropriate, using a left mouse click if this was the case and a right mouse click otherwise. The stimuli were presented along with a fixation cross. All terminal chords of the pieces were cut off at 1,400 ms after onset and faded linearly over the last 150 ms (using Adobe Audition 3.0, Adobe Systems Corp., California, USA). The response screen appeared 1,900 ms after the onset of the terminal chord (stimulus onset) and 500 ms after the disappearance of sound, to avoid EEG contamination by the motor response. The order of presentation was fixed, but there were 2 versions of the task: Direct order with pseudo-randomized presentation, and the reversed order of the latter (in order to cope with possible order effects). Half of the participants of each pitch group completed the direct order and half the reverse order. The task consisted of 5 blocks of 54 pieces, separated by breaks in order to prevent fatigue.

The harmonic transgressions used here, that involve in-key chords that would make perfectly sense if the musical stimuli continued, clearly involve cognitive processing. They represent refined grammatical errors in the musical idiom and are by no means dissonances or out of-key elements that would principally involve perceptual processing. The 2 chords used for transgression are just as consonant (or dissonant, consonance and dissonance are 2 extremes of a continuum) as a tonic chord, both being composed of thirds, sixths, fifths and fourths).

For more comprehensive information on the stimuli and more musical examples we refer to 3 previous publications (Oechslin et al., 2013; James et al., 2017; Jenni et al., 2017).

#### Auditory Selective Attention Test

We created this test based on the figure-ground principle. Participants' task consisted in comparing 2 series of sounds (“melodies”) lasting 500 ms, separated by one-second of brown noise, and reporting whether they were identical by means of a left (“identical”) or right (“different”) mouse click. The series of sounds, the “figures,” consisted of 7 tones, drawn at random (sampling without replacement) from the chosen range: The 12 semitones of the fourth octave of the piano. Together with each tone of the figure, a cluster composed of all 12 semitones played simultaneously (ground), with slightly less amplitude than the figure tones. Therefore, the participants had to compare the series in the presence of background noise, demanding auditory selective attention, even more so as the series were not arranged within a tonality, but randomly drawn. This random arrangement limits the grouping of several notes, hampering memorization of the series. In total, the test comprised 50 different series of 2 times 7 tones, of which each appeared in an “identical” and “different” version resulting in 100 stimuli. In a “different” version, 1 tone among the 7 differed from the first vs. the second series, a semitone higher (23 trials) or lower (27 trials). The modification was applied to one of the middle 5 tones, excluding the first and last one of the series (to avoid facilitating serial position effects). The series of piano-like tones were generated with the “Cool Edit Pro” software (http://cool-edit-pro.soft32.com). Participants received 1 point for each correct answer for a total of 100 points. This test can be succeeded at 100% by means of relative pitch alone, as the participant has to compare interval series.

#### WAIS-IV Digit Span

In order to test auditory short-term memory and working memory, participants accomplished the “digit span” subtest of the Wechsler adult intelligence scale 4th edition (Wechsler, 2011) in direct and reverse order. During these tests, participants listened to spoken series of digits with increasing length (from 2 to 8 digits), and their task consisted in repeating them orally first in direct (testing primarily auditory short-term memory) then in reverse order (testing primarily auditory working memory). Two series of digits (1 for each task), progressively increasing by level of difficulty (length), were presented. Spoken digits were recorded beforehand at a rate of 1 digit per second to guarantee identical conditions for all participants. Participants completed first the direct order, then the reverse order task. They proceeded through the trials until they made 2 successive mistakes at the same level of difficulty. Each correct answer was given 1 point for a total of 16 points per task.

### Loudness/Intensity of the Auditory Stimuli

We normalized all auditory stimuli to an identical amplitude envelope (−5 dB) with the “Cool Edit Pro” software (http://cool-edit-pro.soft32.com). Participants determined the optimal loudness individually during stimulus presentation, therefore we do not report intensity/dB values for the presentation of the stimuli. As the size and shape of the ear cup and canal varies strongly between individuals, inserting the earphones more or less deeply in the ear canal impacts the distance to the eardrum and thus influences perceived loudness. As the participants were all trained musicians, they are used to optimize hearing using earphones.

### EEG Acquisition and Raw Data Processing

Participants were comfortably seated in the EEG room in front of a computer screen with approximate 1 m distance from the eyes. Continuous EEG was acquired using the “Biosemi” system (Amsterdam, The Netherlands) comprising an ActiveTwo amplifier system AD-Box with 64 active AG/AgCL electrodes, sampled at 1,024 Hz in a bandwidth filter of 0–268 Hz. The average of all electrodes was used as an online and offline reference. Meanwhile we recorded electro-oculography (EOG) and applied the voltage difference of 2 horizontal (HEOG) electrodes fixed at the outer canthi side of both eyes, to detect horizontal eye movements. Two additional electrodes were placed above and under the right eye to measure the voltage difference (VEOG) caused by blinks.

Raw data pre-processing was performed using the “BrainVision Analyzer 2.0” software (Brain Products GmbH., Munich, Germany). Offline, data were band-pass filtered between 0.25 and 30 Hz within a Butterworth zero phase filter. An additional 50 Hz notch filter removed noise from the power-line. Finally, given that the low-pass filter was set to 30 Hz, data were down-sampled to 256 Hz to reduce data size, thus processing time. Artifact rejection was applied to exclude voltages above 100 μv and under −100 μv. Independent component analysis was used to remove ocular artifacts and reduce deformation at the edge of the epochs. Finally, a baseline correction of −200 ms to stimulus onset was performed. On average 8.82% (*SD* = 11.38%) of the trials per experimental condition were removed. Data of one AP participant were excluded due to excessive artifacts.

Grand-Averages for the AP group were based on 80.00 ± 13.97 SD epochs for R, 81.00 ± 12.55 SD epochs for T^sub^, and 77.73 ± 15.82 SD epochs for T^app^. For the NAP group, 84.42 ± 5.23 SD epochs were kept for the Grand-Averages for R, 84.25 ± 3.49 SD epochs for T^sub^, and 84.33 ± 4.42 SD epochs for T^app^.

Noisy channels were interpolated, by means of a 3-D spherical spline, for on average 0.34% (*SD* = 1.05%) of the electrodes.

### ERP Analyses

The evoked potentials were analyzed in 3 stages described in detail below.

#### Classical Waveform Analysis

**Figure 2:**
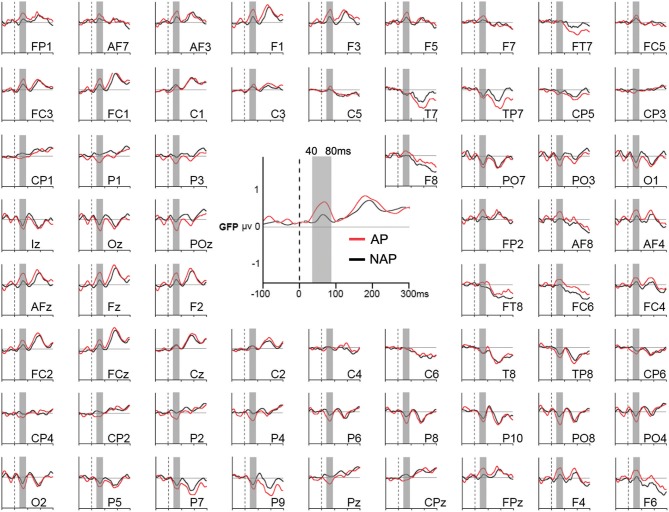
Grand average Global Field Power (GFP; in the middle) and event-related potentials (around) of all 64 electrodes in response to the endings of all musical stimuli for AP (in red) and NAP (in black) participants, independently of the level of musical transgression (R, T^sub^, and T^app^ collapsed). The gray vertical bar corresponds to the 40–80 ms period of statistical testing.

Our main interest lies in the microstate and source analyses that provide more comprehensive information, as they comprise all electrodes. Therefore, as multiple testing over our period of analysis (0–1,000 ms) only showed marginal differences for individual electrodes between AP and NAP for all 3 transgression conditions, we limited our waveform statistics to an analysis of Global Field Power (GFP), all 3 transgression conditions collapsed. GFP provides one single, always positive, reference free value representing the neural response strength throughout the brain, incorporating absolute voltages of all electrodes (Murray et al., 2008). This choice was made because during a very early time period, a generalized effect of pitch group manifested at almost all electrodes in all conditions (Figure 2).

We did not compute difference waves because they do not allow to compute microstates and source estimations. Moreover, as only marginal differences occurred for all 3 transgression conditions between AP and NAP, we collapsed all conditions to study the above mentioned early generalized brain response.

#### Microstate Analysis

Evoked potentials and spontaneous EEG display over time, as scalp voltage topographies remaining relatively stable for tens to hundreds of seconds, and represent microstates of information processing “atoms of thought” (Pascual-Marqui et al., 1995; Murray et al., 2008; Brunet et al., 2011).

Microstate analysis or spatio-temporal ERP analysis consists of 2 stages. During the first stage, a *k*-means cluster analysis applied to the grand-means of both conditions and groups defined the most dominant scalp topographies or microstates (Murray et al., 2008). In order to study robust effects, we chose to group microstates sharing over 92% of variance and lasting more than 20 ms. To define the optimal number of microstates we relied on the cross-validation index (Pascual-Marqui et al., 1995), which minimizes the residual variance, and on a modified “Krzanowski–Lai criterion,” a quality measure for clustering (Michel et al., 2004; Tibshirani and Walther, 2005; Murray et al., 2008; Michel and Brandeis, 2009; Brunet et al., 2011). The resulting microstate series represent an a priori hypothesis, to be statistically verified in the second stage. This second stage consists in “fitting” the microstates back over time across all individual subjects, by means of a spatial correlation analysis, resulting in a parameter of presence, Duration (in ms) and of Global Explained Variance (GEV; in %) of each microstate for a period of interest. Statistical testing of these parameters then verifies differential microstate presence over time comparing groups or conditions.

We started by comparing microstates between the 2 groups (AP vs. NAP) for all 3 conditions of musical transgression (R, T^sub^, and T^app^). One single precocious distinct microstate occurred in all 3 conditions principally in the AP group. No other statistically significant differences occurred as a function of one of the transgression conditions over the further time period of analysis (up to 1,000 ms after stimulus onset). Therefore, we focused on this early microstate dissociating AP and NAP groups, averaging the data over all 3 transgression conditions, and recomputing a microstate analysis only on the Grand-Average ERPs of the 2 groups (R, T^sub^, and T^app^ collapsed). As the aim of this experiment was to study ERP differences as a function of pitch group, we will only focus on the latter analysis in the present paper.

The microstate analysis was performed with the Cartool software, developed by Denis Brunet (http://brainmapping.unige.ch/cartool).

#### Source Analysis

Unlike the direct solution that consists in calculating voltage configurations on the scalp from known underlying sources, the inverse problem of computing the underlying hypothetical sources from the measured voltages on the scalp is not unique or rather “ill-posed.” To deal with this “ill-posed” inverse problem with surface EEG, distributed source analysis should be based on well-defined a priori constraints (Michel et al., 2004; Grech et al., 2008). Our a priori constraints derive from the microstate analysis that allowed determining a period during which distinct microstates appeared in both groups. Differences in scalp topographies over time indicate a change in the underlying generators (Lehmann et al., 1987; Murray et al., 2008), which relates to changes of the brain's functional state (Michel et al., 1999).

As our main interest lies in exploring differences between the AP and NAP group for auditory processing of music, we focused on ERP sources regarding the primary outcome of the microstate analysis, i.e., the early time period during which 2 spatially different microstates appeared in each group. These microstates appeared during the first 100 ms of processing of musical closure, in all experimental conditions. Microstates reflect stable scalp topographies and underlying brain networks, and maps' stability is maximum during the global field power (“GFP”) peak (Koenig et al., 2014). In addition, the signal-to-noise ratio increases with the strength of the GFP (Lehmann et al., 2005). Therefore, we limited the time window of analysis around the GFP peak of the microstate characterizing the AP group (40–80 ms), the NAP group showing no marked GFP peak. This approach of centering source analyses on the GFP peak has been successfully used in different settings (Plomp et al., 2013; James et al., 2015).

We estimated the intracranial distribution (microampere per cubic millimeter, μA/mm^3^) of the averaged ERP for each subject across all experimental conditions over the 40–80 ms period of interest, using an inverse linear solution of depth-weighted minimum norm (Hamalainen and Ilmoniemi, 1994; Michel et al., 2004; “WMN”). The WMN algorithm compensates the tendency of minimum standard algorithms to favor weak and superficial sources by using a weighted matrix (Grech et al., 2008). In combination with a statistical parametric mapping (SPM) approach, consisting of a statistical comparison of the underlying sources between groups, a considerable amount of noise is annihilated. In addition, the SPM method avoids the domination of maxima of the source that are identical in both groups (Michel et al., 2004). The intracranial distribution was calculated within a discrete 3-D grid of 3005 solution points, evenly distributed through the volume of gray matter of the Montreal Neurological Institute (“MNI”) standard brain. By means of a homogeneous transformation that adapted the volume to the most appropriate sphere (“SMAC model”; Spinelli et al., 2000) a 3-shell spherical model was applied to calculate the lead field for the 64 electrodes and the inverse solution based on the constraints of the WMN. Then, to eliminate noise and to compare the groups, we applied the SPM model using 2-sided *t*-tests at each node, by means of the “Statistical Toolbox for Electrical Neuroimaging” (STEN; https://doi.org/10.5281/zenodo.1164037; courtesy of Jean-François Knebel). Results of the SPM consist of stronger current density in certain brain voxels for either group (positive vs. negative *t*-values).

In order to cope with multiple comparisons, we used conservative statistical thresholds and only considered as significant globular clusters of at least 20 contiguous nodes each with a *p*-value of ≤ 0.01 (Knebel et al., 2011; De Meo et al., 2015). The spatial criterion of 20 nodes was determined through the “AlphaSim” software (http://afni.nimh.nih.gov) with a 10 mm FWMH smoothing and 10 mm radius of connected poles, together with the node *p*-value of <0.005 that we applied. Following a bootstrap procedure (10,000 Monte Carlo iterations) a 21-node cluster appeared, with a 0.001 cluster-level probability and a 0.00001 *p*-value corresponding at the node level. Spatial accuracy of these source estimations was thoroughly investigated in the past and showed that these approaches provide similar results compared to fMRI studies, only with a lesser precision range of 1–2 cm (Martuzzi et al., 2009; Plomp et al., 2010; Birot et al., 2014). The WMN source analysis was performed with the Cartool software (brainmapping.unige.ch/cartool).

A common misunderstanding interpreting these analysis techniques is that the source computations are applied to the microstates. The microstate analysis is a separate analysis stage, and also serves to determine the appropriate time periods for the source analysis, that is then applied to the ERPs. The latter are genuine neurophysiological data, whereas the microstate data are the fruit from a statistical cluster analysis.

## Results

For the sake of brevity and transparency statistical results can be found mainly in the tables, and as little as possible in the text. Note that in order to compute effect sizes for our generalized linear mixed model (McCulloch, 2003; Bolker et al., 2009; GLMM) analyses, we used Nakagawa and Schielzeth's method (Nakagawa and Schielzeth, 2013) implemented in the “MuMin” R package. Their method is based on 2 indicators: A marginal (*R*^2^_*m*_) and a conditional (*R*^2^_*c*_) *R*^2^. *R*^2^_*m*_ is the variance explained by the fixed factors, whereas *R*^2^_*c*_ is the variance explained by the entire model (both fixed and random effects).

### Behavioral Results

#### Edinburgh Handedness Inventory

The Edinburgh Handedness Inventory confirmed that all participants were right-handed. Moreover, as verified by a Student independent sample *t*-test comparing the 2 groups (AP vs. NAP) there was no significant difference between the AP (*M* = 79.43; *SD* = 23.45) and NAP (*M* = 86.75, *SD* = 10.80) group concerning handedness level [*t*_(21)_ = 0.98, *P* = 0.34, *d* = 0.40].

#### Absolute Pitch Test

**Figure 3:**
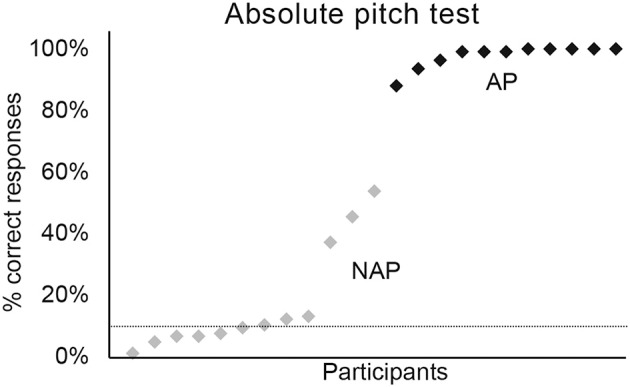
Percentage of correct responses for the absolute pitch test. Graphic representation of individual percentages of correct responses for AP (in black) and NAP participants (in gray).

Concerning the absolute pitch test, we applied a GLMM specifying a binomial model with Group (AP vs. NAP) as fixed factor, and participants' performance as random factor. These analyses demonstrated that the AP group was significantly more accurate than the NAP group (see Figure 3, Table 2). The test yielded a large effect size [*R*^2^_*m*_ = 0.69; (Cohen, 1988; Calin-Jageman, 2018)]. The NAP groups showed a less homogeneous performance than the AP group. Indeed, 9 NAP scored <20% on average and 3 between 30 and 60%. As we intended to oppose true-AP possessors against all other types of pitch perception, the inclusion of 3 quasi-absolute pitch (QAP) possessors seems legitimate (Wilson et al., 2009).

**Table 2:** Percentage of correct responses for the absolute pitch test.

	**AP**	**NAP**				
**Effect**	**Mean accuracy (95% CI)**	**Mean accuracy (95% CI)**	***X*^**2**^**	***P***	***R*^**2**^*_***m***_***	***R*^**2**^*_***c***_***
Group	99% ([97, 100])	12% ([6, 23])	42%	<0.001	0.69	0.80

#### Musical Test

In order to investigate differences in performance at the musical test between AP and NAP participants, we calculated the rater sensitivity index “d-prime” for T^sub^ and T^app^. The d-prime index is a statistic used in signal detection theory (Macmillan and Creelman, 1997), preventing impact of response biases, by comprising both “hits” (correctly detecting that T^sub^ and T^app^ endings are not appropriate) and “false alarms” (incorrectly detecting that R endings are not appropriate) in the calculation of task performance. The d-prime index thus incorporates the responses to the R (regular) endings. A higher d-prime value reflects better discrimination between transgressed and regular endings. Comparison of the d-prime parameter for AP (*M* = 2.28; *SD* = 1.19) and NAP (*M* = 2.22; *SD* = 1.31) participants for T^sub^, using a Student independent sample *t*-test, revealed no significant difference between the groups [*t*_(21)_ = −0.11, *P* = 0.91, *d* = 0.05]. The same analysis showed no significant difference between AP (*M* = 3.77; *SD* = 1.26) and NAP (*M* = 3.82; *SD* = 1.07) participants for T^app^ [*t*_(21)_ = 0.11, *P* = 0.91, *d* = 0.04].

The high d-prime scores in both groups confirm the musical and specifically harmonic skills of our participants. Compared to our previous study comparing expert pianists, amateur pianists and non-musicians (James et al., 2017) mean d-prime scores of the population in the current study for the subtle (difficult to detect) transgressions for the AP (*M* = 2.28; *SD* = 1.19) and NAP group (*M* = 2.22; *SD* = 1.31) are situated in between those of amateur (*M* = 1.54; *SD* = 1.00) and professional pianists (*M* = 4.19; *SD* = 0.78) in James et al. (2017).

#### Auditory Selective Attention Test

GLMMs with the group (AP vs. NAP) as fixed factor and participants' performance as random factor were run. The accuracy at the auditory selective attention test was not significantly different between the AP and NAP group (see Table 3). Two Student one sample *t*-tests demonstrated that the score for the AP group [*t*_(10)_ = 6.25, *P* < 0.001, *d* = 6.26] and NAP group [*t*_(11)_ = 5.36, *P* < 0.001, *d* = 1.55] was significantly different (i.e., above) from chance level (50%). Before analyzing reaction time data (ms), a log base 10 transformation of them was applied to correct for a floor effect and equalize the variances. Results showed that NAP participants were marginally faster than the AP participants (see Table 3).

**Table 3:** Summary of the Group results (AP vs. NAP) at the auditory selective attention test and WAIS IV direct and indirect order subtests.

	**AP**	**NAP**				
	**Mean accuracy (95% CI)**	**Mean accuracy (95% CI)**	***X*^**2**^**	***P***	***R*^**2**^*_***m***_***	***R*^**2**^*_***c***_***
Auditory selective attention test	66% ([61; 71])	66% ([60; 71])	0.01	0.91	<0.001	0.30
WAIS IV direct order	80% ([72; 86])	78% ([71; 84])	0.12	0.73	<0.001	<0.001
WAIS IV indirect order	78% ([70; 84])	77% ([70; 83])	0.01	0.91	<0.001	<0.001
	**Mean RT (ms)-log10** **(95% CI)**	**Mean RT (ms)-log10** **(95% CI)**	***X***^**2**^	***P***	***R***^**2**^*_***m***_*	***R***^**2**^*_***c***_*
Auditory selective attention test	6.23 ([6.04; 6.42])	5.97 ([5.78; 6.16])	3.34	0.07	0.03	0.19

#### WAIS-IV Digit Span

A GLMM with the group (AP vs. NAP) as fixed factor and participants' performance as random factor was performed on the scores of the direct order digit span subtest. The analysis revealed that AP participants' scores were not significantly different from those of the NAP participants (see Table 3). There was again no significant difference between the AP and NAP group concerning the indirect order digit span subtest (see Table 3).

### EEG Results

#### Classical Waveform Analysis

A 2-tailed unpaired *t*-test comparing mean GFP over the time window 40–80 ms between AP and NAP groups, all 3 transgression conditions collapsed (i.e., R, T^sub^, and T^app^), yielded a significant result, with greater strength for AP (*M* = 0.71 μV*, SD* = 0.18) than for NAP (*M* = 0.49 μV*, SD* = 0.23) participants [*t*_(21)_ = 2.47*, P* = 0.02*, d* = 1.07]. With *d* = 1.07, the effect size is large (Cohen, 1988; Calin-Jageman, 2018).

As we collapsed all 3 conditions (R, T^sub^ and T^app^), the GFP Grand-Averages of AP and NAP were based on 239.18 ± 40.19 SD epochs for AP participants, and 253.75 ± 13.20 SD epochs for NAP participants.

#### Microstate Analysis

**Figure 4:**
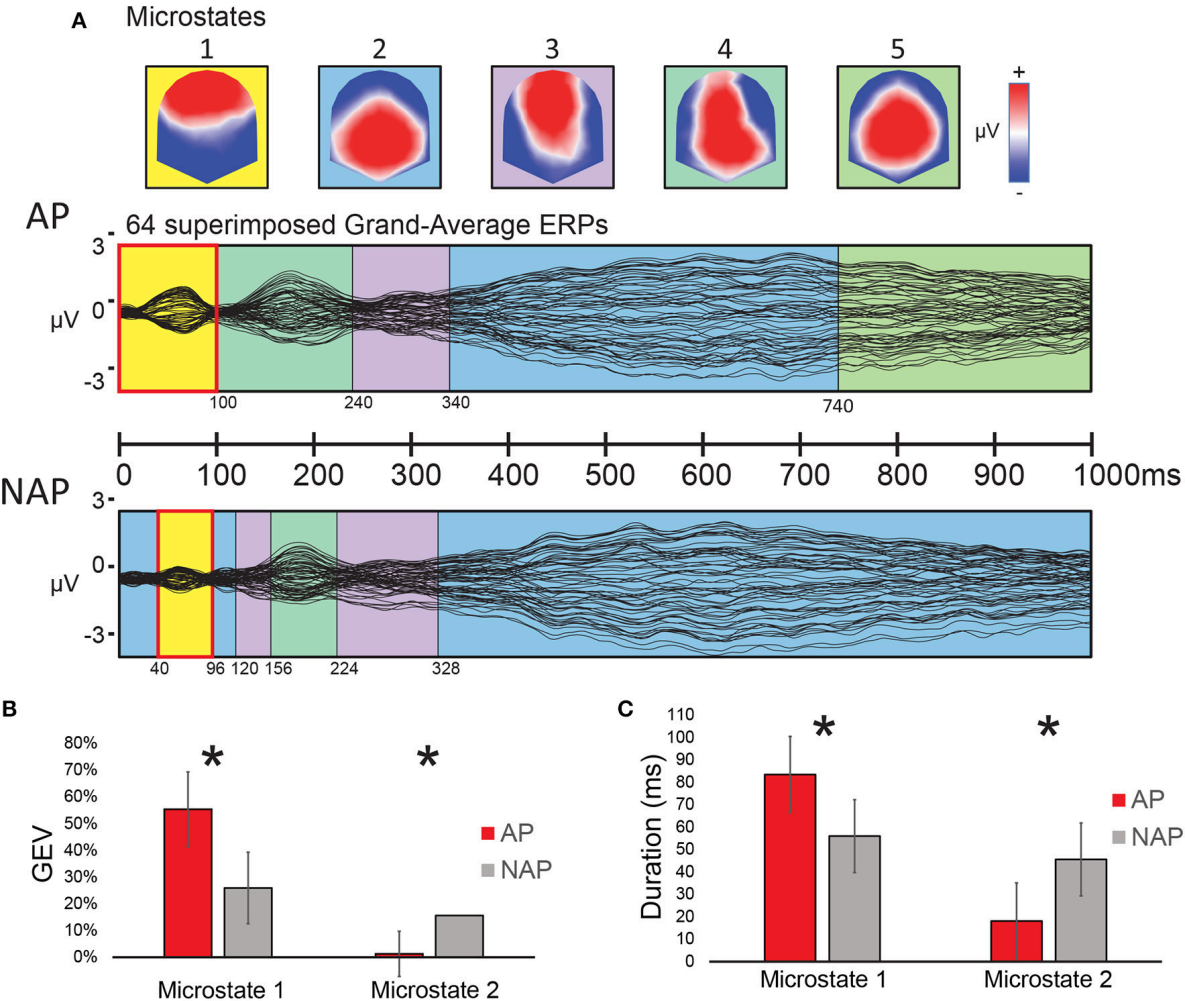
Results of the microstate analysis on Grand Average ERP responses to the musical test (0–100 ms), all transgression conditions collapsed, for AP and NAP participants. **(A)** Top panel: voltage configurations of the 5 microstates resulting from the microstate analysis; Middle and bottom panels: microstates marked in color-code on the superimposed Grand-average ERP waveforms of all 64 electrodes for AP (middle panel) and NAP (bottom panel). **(B,C)** Fitting results (0–100 ms). **(B)** Global explained variance (GEV) by group for microstates 1 and 2. **(C)** Duration by group for the same microstates. Asterisks indicate significant differences between the groups at *P* < 0.05.

A spatio-temporal analysis between 0 and 100 ms on the Grand-Average ERPs of the 2 groups (stage 1, see “Classical Waveform Analysis”) yielded a solution with 5 stable microstate map configurations over time, independently of experimental condition (i.e., R, T^sub^, and T^app^ collapsed). The series of microstates appearing over time are depicted in Figure 4A. These 5 microstates explained 96% of the ERP data variance. Visual inspection of this microstate series at the grand mean level showed a similar sequence of microstates for both groups except from 0 to 156 ms and from 740 to 1,000 ms. Only for the early period significant statistical differences could be found. Between 0 and 100 ms after stimulus onset, a frontal positivity (microstate 1) characterized the AP participants, whereas for NAP participants, both microstate 1 and microstate 2, the latter showing posterior positivity, occurred. Interestingly microstates 1 and 2 manifested almost opposite voltage configurations. Statistical analyses (stage 2, “fitting” see “Microstate Analysis”) on the microstate parameters Duration and GEV between 0 to 100 ms, a period of difference also observed in our ERP data (see Figure 2), reached significance. Because the microstate Durations were not normally distributed, we opted for non-parametric testing for both parameters GEV and Duration. A Kruskal-Wallis statistical comparison based on the fitting of these maps into the individual data showed a significant difference of the parameter GEV between the groups (AP vs. NAP) for microstate 1 [*H*(1, *N* = 23) = 4.91, *P* < 0.05, *d* = 0.95] and microstate 2 [*H*(1, *N* = 23) = 4.46, *P* < 0.05, *d* = 0.89; see Figure 4B]. A significant difference between groups was also found for Duration in ms for microstate 1 [*H*(1, *N* = 23) = 4.34, *P* < 0.05, *d* = 0.87] and microstate 2 [*H*(1, *N* = 23) = 4.34, *P* < 0.05, *d* = 0.87; see Figure 4C]. These results disclosed that, in the 0–100 ms window of interest, microstate 1 principally characterized AP participants, whereas microstate 2 characterized NAP participants. Effect sizes, varying between *d* = 0.87 and *d* = 0.95 are large (Cohen, 1988; Calin-Jageman, 2018).

#### Source Analysis

The a priori constraint for determining the time period for comparing AP and NAP participants for underlying brain sources of the surface EEG was the simultaneous occurrence of 2 distinct microstates (1 and 2; see Figure 4A) in both groups in all experimental conditions in the first 100 ms after stimulus onset. These microstates derive from the microstate analysis comparing the 2 groups, over all experimental conditions (R, T^sub^, and T^app^ collapsed). The scalp configurations of these 2 microstates representing each essentially 1 pitch group, displayed quasi-opposite voltage configurations at the scalp level, with a clear GFP peak for AP possessors for microstate 1. Therefore, this time period provides a strong precursor for computing inverse solutions. To cancel out as much noise as possible, we centered our analysis around the GFP peak of microstate 1 in the AP group (40–80 ms), and applied SPM, comparing the groups with 2-tailed *t*-tests for all 3,005 nodes of the 3-D grid, using the STEN software.

**Figure 5:**
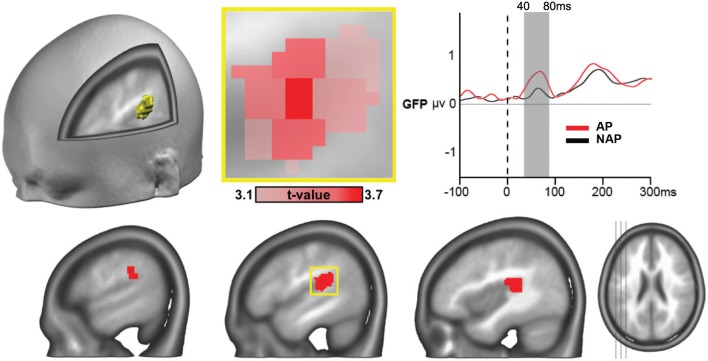
ERP source analyses showing stronger mean brain current density for AP vs. NAP pianists, all musical conditions collapsed, over the 40–80 ms period, in right secondary auditory areas at the temporo-parietal junction (*P* < 0.01, > 20 contiguous nodes). The peak voxel (area of occurrence framed in yellow at the lower panel) occurred in the left posterior superior temporal gyrus [*t*_(21)_ = 3.65, *P* < 0.002, *d* = 0.76; see close-up, in upper middle panel). Right top panel: GFP curves for AP (in red) and NAP (in black) with significant difference in strength between 40 and 80 ms.

One single 21-node globular cluster passed our statistical thresholds (see section “Source Analysis”) characterizing the AP group (positive *t*-values), in left secondary auditory areas located around the temporo-parietal junction, encompassing part of the planum temporale (Figure 5). The peak *t*-value was found in the gray matter of the left posterior superior temporal gyrus [*t*_(21)_ = 3.65, *P* < 0.002, *d* = 0.76; Figure 5, middle upper panel], the other areas composing the cluster comprised parts of the supramarginal gyrus and the rolandic operculum. With *d* = 0.76 we report a close to large effect size here (Cohen, 1988; Calin-Jageman, 2018).

## Discussion

The key and novel finding of this study is that a precocious ERP microstate, peaking as early as 60 ms after closure onset in expressive music, dissociated AP pianists from NAP pianists, in response to all musical stimuli, independently of the harmonic congruence of the musical closure (partially confirming Hypothesis 2). Later more cognitive cerebral processing of harmonic transgressions in music turned out alike in AP and NAP pianists. Identical rater sensitivity (d-prime) for musical transgressions and equal STM and WM scores underpin this observation.

Despite the absence of a behavioral difference for the harmonic transgression test, the robust behavioral difference for the AP test evidences that we are dealing with 2 intrinsically distinct populations, that process the musical stimuli differently, resulting, however, in a similar level of transgression detection proficiency.

According to the 2-level model of AP functioning (Levitin, 1994; Zatorre, 2003; Levitin and Rogers, 2005), this precocious microstate involves the perceptual representation of pitches in long-term memory and not the semantic labeling. Given its very early appearance, this stage of information processing apparently consists in a highly automatized pitch analysis capacity, which can be dissociated from later more cognitive processing of music. Our source analyses revealed that this microstate concurred in AP musicians with involvement of left secondary auditory areas at the temporo-parietal junction, comprising the planum temporale, corresponding to those described in the literature distinguishing AP possessors (Schlaug et al., 1995; Ohnishi et al., 2001; Jancke et al., 2012; partially confirming Hypothesis 4). Moreover, Elmer et al. (2015) hypothesized that specifically the left planum temporale (PT), that is part of the cluster the current study localized, fosters the first perceptual component of AP according to the 2-level model, matching incoming spectrotemporal patterns with a template (Ohnishi et al., 2001). These left auditory related areas may function as a “hub of information flow” toward inferofrontal areas (Elmer et al., 2015, p. 369).

These results also constitute another confirmation of the validity of source analysis from scalp EEG (Plomp et al., 2010; Birot et al., 2014), while adding a temporal precision that other imaging techniques cannot provide (Michel et al., 2004; Meyer-Lindenberg, 2010; Michel and Murray, 2012).

To the best of our knowledge, our study is the first to highlight such an early difference between AP and NAP participants. The earliest differences reported in previous studies concerned the N150 ERP component (Itoh et al., 2005), and the MEG M100 (Hirata et al., 1999). Given the component's time of occurrence, and the fact that it appears much earlier than the MMN (mismatch negativity) which reflects pitch memorizing, and the P3a, the latter probably reflecting the labeling process (Rogenmoser et al., 2015), we suppose that it highlights an automatic and unconscious sensorial process.

The fact that we were able to identify specific sources underlying AP function in left secondary auditory areas, including the PT at this early point in time, is noteworthy. The assumed high degree of automation of this special pitch processing and identification capacity is strongly supported by this result. This processing stage thus precedes cognitive processing of complex music.

The literature showed that rather pruning in the right PT than development of the left PT explains the observed left-right PT asymmetry in AP possessors (Keenan et al., 2001). This underdevelopment of the right PT then favors functional dominance of the left PT during early development in AP individuals. The here observed pitch processing likely performed by the left PT and surrounding areas undoubtedly represents only part of the AP faculty, i.e., the acoustical part. Left frontal areas, more precisely the left posterior dorsolateral prefrontal areas, may carry the “linguistic labeling” of the acoustically identified pitches (Zatorre et al., 1998; Ohnishi et al., 2001). Our study did not highlight any frontal sources, which can be explained by the SPM analysis technique that annihilates all common activations in both groups and only shows differences. The NAP musicians in the current study, that are also highly trained musicians, likely also rely on left dorsolateral prefrontal areas when making relative pitch judgments of chords, relevant for the task in the current experiment (Zatorre et al., 1998; James et al., 2017). Likewise, differences in right auditory cortex activation did not reach statistical significance either as AP and NAP participants probably both relied on these brain areas for chord processing.

Two studies investigated brain connectivity in AP possessors. Enhanced structural brain connectivity in AP people was described by Loui et al. (2011) and involved tracts connecting more strongly the left superior temporal gyrus with the left middle temporal gyrus. Volumes of these tracts predicted AP performance. Furthermore these authors observed hyperconnectivity in bilateral superior temporal lobe structures in AP possesors. A recent study by Brauchli et al. (2019) observed enhanced fine-grained functional connectivity in the left auditory cortex in AP musicians using multi-voxel pattern analysis. These studies do not refute our findings.

Our study deviates however from 2 important publications in the domain of comparison between AP and non-AP musicians. First, we did not find right Heschl's involvement in AP processing like Wengenroth et al. (2014). This may be explained in the first place by the different time window studied with fMRI, not allowing to detect a precocious effect like we could in the current study. Secondly, the AP definition slightly differed, as the authors applied an innovative AP test, excluding any RP involvement, moreover, also accepting whole tone precision -albeit with weighted scoring. In the test used here, relative pitch perception is involved. Additionally, in Wengenroth et al. (2014), tones and not expressive music served as stimuli, and the participants played different instruments. As stated before, observed differences concerning AP may be explained by the diversity of its definitions, and by the absence of consensus concerning its measurement (Hou et al., 2016).

Within a passive listening Oddball paradigm, Rogenmoser et al. (2015) found identical MMN responses in AP and NAP musicians but different P3a responses, concluding that early auditory processing did not differ between the groups, but later more cognitive processing did, with AP showing smaller P3a amplitudes. We report opposite results, with a very early difference arising from secondary auditory areas at 60 ms and no later differences in dorsolateral prefrontal areas. This difference in observations may be explained by the different stimuli used, and also by the criteria for AP. Rogenmoser et al. (2015) tested their participants on out-of-tune tones in a passive listening Oddball paradigm. In that case the automatic searching for the correct label seems triggered in AP possessors, because out of tune notes may induce doubt on the exact pitch of the note, despite the passive listening, as AP people process pitch automatically and involuntary. In our active harmonic transgression task, searching for the correct label is not prominent, searching for the correct syntactical function of the chord is, implicating also relative pitch processing. Moreover, in Rogenmoser et al. (2015), AP possessors provided at least 40% of correct responses in the AP test, vs. 90% in our study. So several of our NAP participants would have been categorized as AP in their study and their results did not restrain to true-absolute pitch processors. Finally, in a replication study of Rogenmoser et al. (2015), performed by Greber et al. (2018) on a larger sample of musicians, the findings on a weaker P3a in AP possessors were not confirmed.

An earlier EEG study by our research group compared participants with 3 levels of training intensity for the 3 transgression conditions (James et al., 2017). In that study, non-musicians, amateur pianists and expert pianists showed pronounced differences in ERP responses and source estimations between the 3 transgression conditions. Interestingly, in the current experiment with 2 groups not differing in training intensity, no such differences were found, although we expected this initially. As, moreover, no differences manifested for transgression detection at the behavioral level, these results suggest that no important differences exist between equally trained AP and NAP pianists for cognitive music processing as involved in our task (infirming Hypothesis 1).

We initially hypothesized that AP participants would perform better than NAP participants at auditory STM and WM tasks. Concerning STM, we expected to replicate Deutsch and Dooley's results (2013), who used similar stimuli and task: Series of digits that participants had to repeat in direct order, for which they observed an advantage for AP participants. In contrast, we observed similar scores in both groups for STM and WM (partially infirming Hypothesis 3). These contradictory results may be explained by differential recruiting of participants and the tests used. Deutsch and Dooley tested 7 APs against 20 NAPs. The instruments played were not specified, thus probably varied. Handedness was not reported. In the current experiment groups were of similar size and composed of right-handed pianists only. Finally, we used the WAIS-IV normed subtests to assess STM and WM, whereas Deutsch and Dooley used a test that they created for their experiment. Interestingly, Benassi-Werke et al. (2012) using the direct and indirect order digit span subtests of the WAIS-III like in the current experiment, did not report any significant differences either between two groups of professional singers with and without AP and a group of amateur NAP singers. However, when testing on tones instead of digits, the AP group performed significantly better. STM and WM advantages possibly only manifest clearly when the test material consists of tones.

As already explained in the introduction, possessing AP may also yield disadvantages in musical contexts. Although not present at the level of correct responses, AP participants responded marginally later than NAP participants in our in-house auditory selective attention test (partially confirming Hypothesis 3). This may be explained by the fact that because AP possessors automatically process all pitches they hear (Akiva-Kabiri and Henik 2012), they are unable to ignore the “ground,” therefore slowing down the processing of relevant information for the task (comparing the tone series). Defining and confirming more precisely domains of disadvantage of the AP skill represents an interesting domain for further research.

## Limitations

As the study population consisted exclusively of pianists, all conclusions also restrain to this particular population. Pianists perform highly complex polyphone scores as compared to melody instrumentalists like string or wind players. Therefore, NAP pianists are also very proficient in the analyses of chords, which may partially explain why no differences occurred for music syntactic processing as a function of AP.

Concerning source computations, since we did not use digitized electrode positions nor individual MRIs, our source localizations are approximately 10% less sensitive (Brodbeck et al., 2011; James et al., 2017) relative to studies that used such individualized techniques (Birot et al., 2014; Megevand et al., 2014; Plomp et al., 2015).

Finally, the sample size of this study is relatively small. From our point of view there is no reason to suspect that the current study is underpowered due to a lack of sensitivity. As we report large effect sizes for all results (absolute pitch test, classical waveform analysis on GFP, microstate analysis and source analyis), we fulfill the recommendations of Whitehead et al. (2016), with at least 10 participants per group for studies with large effect sizes, in order to obtain 90% power and two-sided 5% significance.

## Conclusions

To summarize, the key and novel finding of this study is the revelation of a precocious microstate (peaking around 60 ms after stimulus onset) characterizing AP participants in response to musical closure in complex polyphone music. Given its very early occurrence and underlying brain sources at this time point in secondary auditory areas, this stage of information processing clearly involves the acoustical features of AP functioning. Specific cerebral sources for AP possessors contributing to this microstate were localized in secondary auditory regions at the temporo-parietal junction, including parts of the PT. These brain areas correspond to those described in the literature to distinguish AP possessors at the anatomical and functional level (Schlaug et al., 1995; Keenan et al., 2001; Itoh et al., 2005). In contrast, AP did not facilitate later musical cognition or general cognition. These findings therefore demystify to some extent the incrusted beliefs on the major impact of AP on musical skills.

We consider our findings particularly interesting, because it is the first study to compare such homogeneous populations for investigating AP. Indeed, as AP and NAP participants in our study did not differ for age, age of onset of musical practice, total number of years of music training, gender, instrumental level and socio-economic status, they exclusively differed for AP skill, making our results stand out from previous research in the domain.

## Author Contributions

SYC, NV, DG, and CEJ conceived and designed the study. NV recruited the participants. SYC and NV welcomed the participants and administered the EEG recordings. SYC, NV, and CEJ analyzed the data, designed the figures and wrote the main manuscript text. All authors were involved in the discussions about the interpretation of the results and the proofreading of the manuscript.

### Conflict of Interest Statement

The authors declare that the research was conducted in the absence of any commercial or financial relationships that could be construed as a potential conflict of interest.

## Supplementary Material

Audio 1 Regular closure (degree I).

Audio 2 Subtle transgression of closure (T^sub^, degree I^6^).

Audio 3 Apparent transgression of closure (T^app^, degree IV^6^).
